# Comparisons of short-term and survival outcomes of laparoscopy-assisted versus open total gastrectomy for gastric cancer patients

**DOI:** 10.18632/oncotarget.17019

**Published:** 2017-04-10

**Authors:** Xin-Zu Chen, Shao-Yong Wang, Yin-Su Wang, Zi-Han Jiang, Wei-Han Zhang, Kai Liu, Kun Yang, Xiao-Long Chen, Lin-Yong Zhao, Meng Qiu, Hong-Feng Gou, Zong-Guang Zhou, Jian-Kun Hu

**Affiliations:** ^1^ Department of Gastrointestinal Surgery, West China Hospital, Sichuan University, Chengdu, China; ^2^ Institute of Gastric Cancer, State Key Laboratory of Biotherapy/Collaborative Innovation Center of Biotherapy, West China Hospital, Sichuan University, Chengdu, China; ^3^ Department of Gastrointestinal Surgery, Guizhou Provincial People's Hospital, Guiyang, China; ^4^ Faculty of Medicine, West China Medical School, Sichuan University, Chengdu, China; ^5^ Department of Medical Oncology, Cancer Center, State Key Laboratory of Biotherapy, West China Hospital, Sichuan University, Chengdu, China; ^6^ Institute of Digestive Surgery, State Key Laboratory of Biotherapy, West China Hospital, Sichuan University, Chengdu, China

**Keywords:** gastric cancer, laparoscopy, gastrectomy, survival, surgical oncology

## Abstract

**Objectives:**

The safety and surgical oncology of laparoscopy-assisted total gastrectomy (LATG) remain inconclusive and challenging. This study aimed to compare the short-term and long-term outcomes between LATG and open total gastrectomy (OTG) procedures.

**Results:**

In the all-included analyses, there were 69 patients in the LATG group and 268 in the OTG group. LATG was as safe as OTG without increasing postoperative morbidity and mortality. Stage imbalance might introduce differences in the numbers of harvested lymph nodes in LATG (34.4 ± 12.0) and OTG (40.9 ± 16.9), whereas 95.7% of patients underwent D2/D2+ dissection during the LATG procedure. After a median 31 months of follow-up, the overall survival outcomes were comparable between the LATG and OTG procedures (HR = 1.16, 95% CI 0.68–1.97). Sensitivity analysis found comparable node retrieval and stage-specific or treatment-specific overall survival.

**Materials and Methods:**

A retrospective case-control study was conducted among gastric cancer patients who underwent either LATG or OTG with curative intention between June 2006 and December 2015. Data retrieval was based on the Surgical Gastric Cancer Patient Registry in the West China Hospital. The primary outcome was overall survival. The secondary outcomes were postoperative complication incidence and severity, operation duration, blood loss, number of harvested lymph nodes, and postoperative hospital stay. Matched pairwise case-control comparisons were performed as a sensitivity analysis.

**Conclusions:**

LATG by experienced surgeons possibly has comparable short-term surgical outcomes and long-term survival outcomes compared with OTG for gastric cancer patients. However, high-quality RCTs are necessary before confirmative judgment and recommendation as an optional treatment in general practice.

## INTRODUCTION

Gastric cancer is one of the common causes of cancer-related death worldwide [1]. In China, gastric cancer has similarly caused a heavy health burden for decades given that greater than 80% of patients have locally advanced or metastatic disease [2–5]. En bloc resection is the only curative treatment, but some surgical patients experience recurrent disease despite curative intention [1]. Therefore, the surgical oncologic outcome for gastric cancer remains concerning, and a new surgical technique needs to be assessed. Laparoscopic gastric cancer surgery was first performed in 1991 [6], and the laparoscopic gastrectomy was first introduced to mainland China in 1993 [7]. Currently, laparoscopic surgery for gastric cancer has become increasingly popular not only in eastern countries but also in western countries given its minimally invasive nature [8, 9].

In eastern countries, several multicenter trials were conducted or launched to evaluate laparoscopic gastric cancer surgery. Laparoscopic surgery was feasible and even safer than open surgery in distal gastrectomy among early gastric cancer patients in the KLASS-01 trial [10]. Additionally, laparoscopic distal gastrectomy with D2 lymphadenectomy was also feasible and safe among locally advanced gastric cancer patients in the CLASS-01 trial [11]. Some retrospective evidence and meta-analysis demonstrated that the long-term survival outcomes might be comparable between laparoscopic and open gastrectomy for either early or locally advanced gastric cancer [12–14]. Therefore, given the increasing incidence of upper gastric and esophagogastric junctional carcinoma in eastern countries [15, 16], the laparoscopic technique has been expanded to total gastrectomy for gastric cancer by an increasing number of gastrointestinal surgeons. Laparoscopy-assisted total gastrectomy (LATG) is the most common used minimally invasive technique in the aspect of total gastrectomy. However, compared with laparoscopic distal gastrectomy, LATG may be technically more complex and difficult. Therefore, the feasibility, safety and surgical oncology of LATG remain inconclusive given the inadequacy of clinical evidence. Before performing a randomized controlled trial (RCT), we aimed to compare LATG and open total gastrectomy (OTG) procedures in a retrospective study.

## RESULTS

### Patients and follow-up

**Figure 1:**
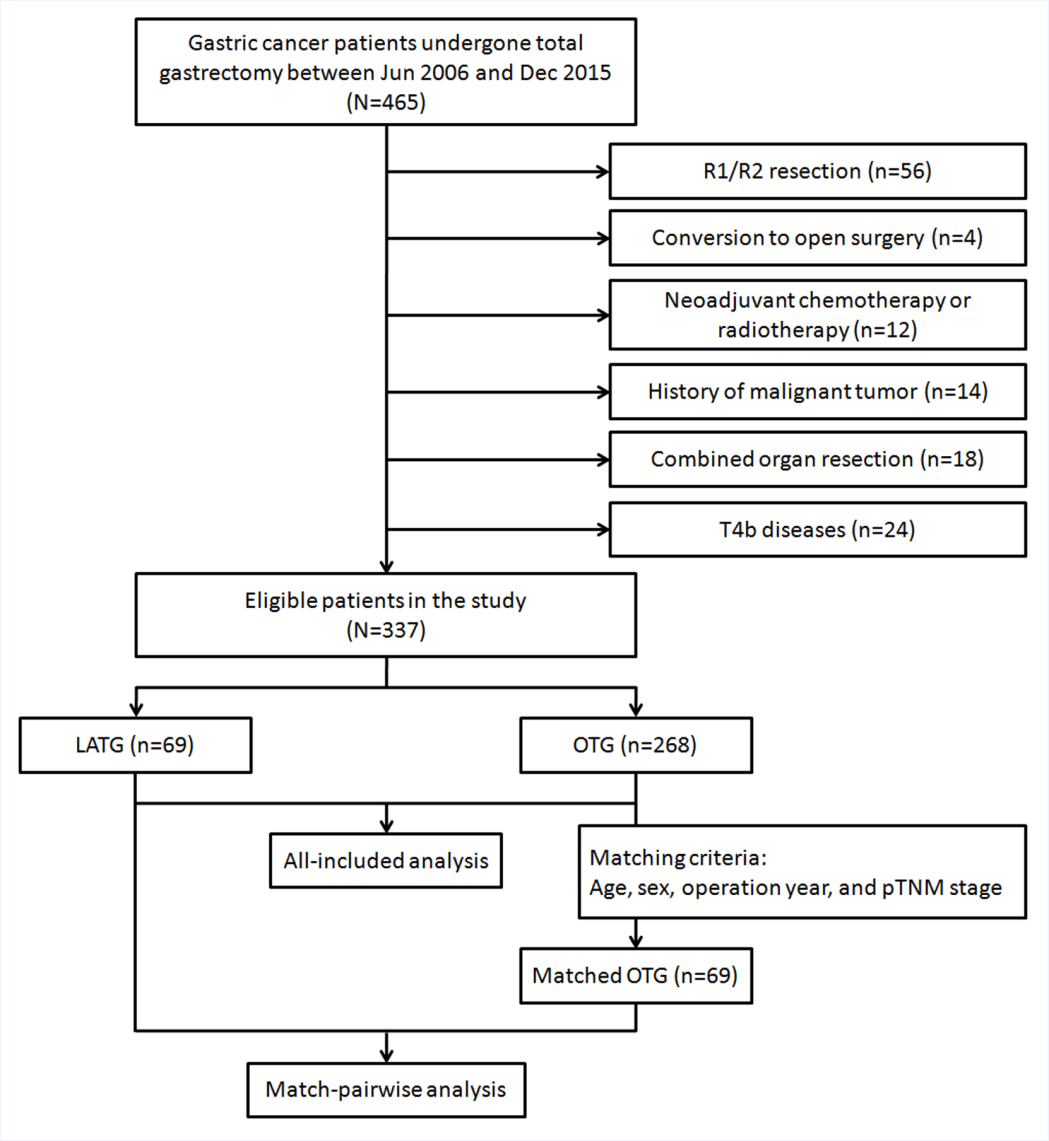
Flow chart of patient selection and matching

A total of 337 patients treated from Jun 2006 to Dec 2015 were eligible for the present study (Figure 1). In the all-included analyses, there were 69 patients in the LATG group and 268 in the OTG group. In the sensitivity analysis, 69 pairwise patients were analyzed in each group. Additionally, given the preoperative intention of the LATG procedure, 4 out of 76 (5.3%) patients experienced conversion to open surgery based on difficulty in completion of D2 dissection, and 3 (3.9%) patients had peritoneal seeding and were unfit for surgery.

Second, in the all-included survival analyses, 270 patients (59 in LATG and 211 in OTG) were identified from Jun 2006 to Dec 2014 with an overall follow-up rate of 97.4% (7 lost) and a median of 31 months (interquartile 17–48 months). In the sensitivity analysis, 59 pairwise patients were analyzed with an overall follow-up rate of 96.6% (4 lost) and a median of 48 months (interquartile 24–63 months).

### Clinicopathological features

In the OTG group, the patients were older and had higher body mass index (BMI) and larger tumor size compared with the LATG group (Table 1). Moreover, patients in the LATG group had earlier disease stages compared with those in the OTG group, especially more N0 and stage I diseases. Therefore, the pathological TNM stage was matched in the sensitivity analysis to reduce selection bias.

**Table 1:** The clinicopathological features of analyzed patients

	All patients		*P* value	Match-pairwise patients		*P* value
	OTG	LATG		OTG	LATG	
	*N* = 268 (%)	*N* = 69 (%)		*N* = 69 (%)	*N* = 69 (%)	
Age (years)*	60.7 ± 9.9	57.1 ± 10.1	0.005	60.5 ± 9.3	57.1 ± 10.1	0.035
Sex (male)	209 (78.0)	58 (84.1)	0.267	58 (84.1)		Matched
BMI (kg/m^2^)*	22.6 ± 3.0	21.1 ± 2.1	0.001	23.0 ± 3.0	21.1 ± 2.1	< 0.001
Tumor size (cm)*	6.0 ± 2.5	4.7 ± 2.1	< 0.001	5.7 ± 2.3	4.7 ± 2.1	0.010
Tumor site			0.155			0.552
U/EGJA	172 (64.2)	41 (59.4)		37 (53.6)	41 (59.4)	
M	49 (18.3)	20 (29.0)		18 (26.1)	20 (29.0)	
ML	44 (16.4)	7 (10.1)		13 (18.8)	7 (10.1)	
UML	3 (1.1)	1 (1.4)		1 (1.4)	1 (1.4)	
Macroscopic type			0.098			0.187
Type 0	16 (6.0)	7 (10.1)		9 (13.0)	7 (10.1)	
Type 1	4 (1.5)	1 (1.4)		2 (2.9)	1 (1.4)	
Type 2	120 (44.8)	36 (52.2)		29 (42.0)	36 (52.2)	
Type 3	103 (38.4)	24 (34.8)		22 (31.9)	24 (34.8)	
Type 4	25 (9.3)	1 (1.4)		7 (10.1)	1 (1.4)	
Differentiation degree			0.402			1.000
G1/G2	43 (16.0)	14 (20.3)		14 (20.3)	14 (20.3)	
G3/G4	225 (84.0)	55 (79.7)		55 (79.7)	55 (79.7)	
T stage*			0.407			0.928
T1	16 (6.0)	7 (10.1)		9 (13.0)	7 (10.1)	
T2	30 (11.2)	12 (17.4)		10 (14.5)	12 (17.4)	
T3	62 (23.1)	10 (14.5)		10 (14.5)	10 (14.5)	
T4a	160 (59.7)	40 (58.0)		40 (58.0)	40 (58.0)	
N stage*			< 0.001			0.191
N0	53 (19.8)	24 (34.8)		22 (31.9)	24 (34.8)	
N1	50 (18.7)	16 (23.2)		12 (17.4)	16 (23.2)	
N2	56 (20.9)	16 (23.2)		11 (15.9)	16 (23.2)	
N3	109 (40.7)	13 (18.8)		24 (34.8)	13 (18.8)	
M stage*			0.127			1.000
M0	252 (94.0)	68 (98.6)		68 (98.6)	68 (98.6)	
M1	16 (6.0)	1 (1.4)		1 (1.4)	1 (1.4)	
TNM stage*			0.011			Matched
Stage I	24 (9.0)	14 (20.3)		14 (20.3)		
Stage II	59 (22.0)	16 (23.2)		16 (23.2)		
Stage III	169 (63.1)	38 (55.1)		38 (55.1)		
Stage IV^#^	16 (6.0)	1 (1.4)		1 (1.4)		
No. of metastatic lymph nodes*	7.7 ± 9.6	3.9 ± 5.5	< 0.001	5.7 ± 7.0	3.9 ± 5.5	0.176
Perineural invasion	42 (15.7)	11 (15.9)	0.956	6 (8.7)	11 (15.9)	0.195
Vessel invasion	44 (16.4)	11 (15.9)	0.924	13 (18.8)	11 (15.9)	0.653
With adjuvant chemotherapy	137 (51.1)	24 (34.8)	0.015	25 (36.2)	24 (34.8)	0.859

*Mann-Whitney *U* test

^#^Metastatic nodes beyond the region of D2 lymphadenectomy.

### Short-term surgical results

In the all-included comparisons, the LATG group had a slightly reduced postoperative hospital stay (*p* = 0.011) but with a median difference of only one day (Table 2). The risks of overall postoperative or specific postoperative complications were not significantly different between the OTG and LATG groups. The severity of postoperative complications was assessed by Clavien-Dindo classification and was not significantly different between the two groups. No deaths (Clavien-Dindo grade 5) were noted among the present observations.

**Table 2:** Comparison on short-term surgical outcomes between LATG and OTG groups

	All patients		*P* value	Match-pairwise patients		*P* value
	OTG	LATG		OTG	LATG	
D2/D2+ lymphadenectomy	265 (98.9%)	66 (95.7%)	0.093	68 (98.6%)	66 (95.7%)	0.334
No. of harvested lymph nodes*	40.9 ± 16.9	34.4 ± 12.0	0.004	34.7 ± 16.0	34.4 ± 12.0	0.777
≥15 nodes	262 (97.8%)	66 (95.7%)	0.333	63 (91.3%)	66 (95.7%)	0.301
≥25 nodes	233 (86.9%)	54 (78.3%)	0.070	52 (75.4%)	54 (78.3%)	0.687
Reconstruction pattern			0.038			0.080
RY EJS	252 (94.0%)	69 (100%)		66 (95.7%)	69 (100%)	
RY EJS with pouch	16 (6.0%)	0		3 (4.3%)	0	
Operation duration (min)*	255.6 ± 41.5	291.5 ± 54.1	< 0.001	259.8 ± 36.9	291.5 ± 54.1	< 0.001
Blood loss (ml)*	131.5 ± 82.7	136.7 ± 77.8	0.915	146.1 ± 88.8	136.7 ± 77.8	0.620
Postoperative hospital stay (days)	10 (9–12)	9 (9–10)	0.011	10 (9–12)	9 (9–10)	0.097
Postoperative overall complications	62 (23.1%)	12 (17.4%)	0.306	8 (11.6%)	12 (17.4%)	0.336
Pattern of complications						
Ileus	4	0	0.565	0	0	1.000
Postoperative pulmonary complications	34	7	0.566	4	7	0.352
Intraabdominal infection	11	3	0.928	3	3	1.000
Superfical surgical site infection	4	1	0.979	0	1	0.498
Intraperitoneal hemorrhage	4	0	0.565	1	0	0.498
Anastomotic leakage	1	1	0.336	0	1	0.498
Pancreatic fistula	4	0	0.565	0	0	1.000
Clavien-Dindo classification*, ^#^			0.212			0.158
Grade 1	36 (58.1%)	8 (72.7%)		3 (37.5%)	8 (72.7%)	
Grade 2	12 (19.4%)	3 (27.3%)		3 (37.5%)	3 (27.3%)	
Grade 3	9 (14.5%)	0 (0)		2 (25.0%)	0 (0)	
Grade 4	5 (8.1%)	0 (0)		0	0 (0)	

Abbreviations: EJS, esophagojejunostomy; RY, Roux-en-Y.

*Mann-Whitney *U* test; ^#^None of grade 5 events.

**Figure 2:**
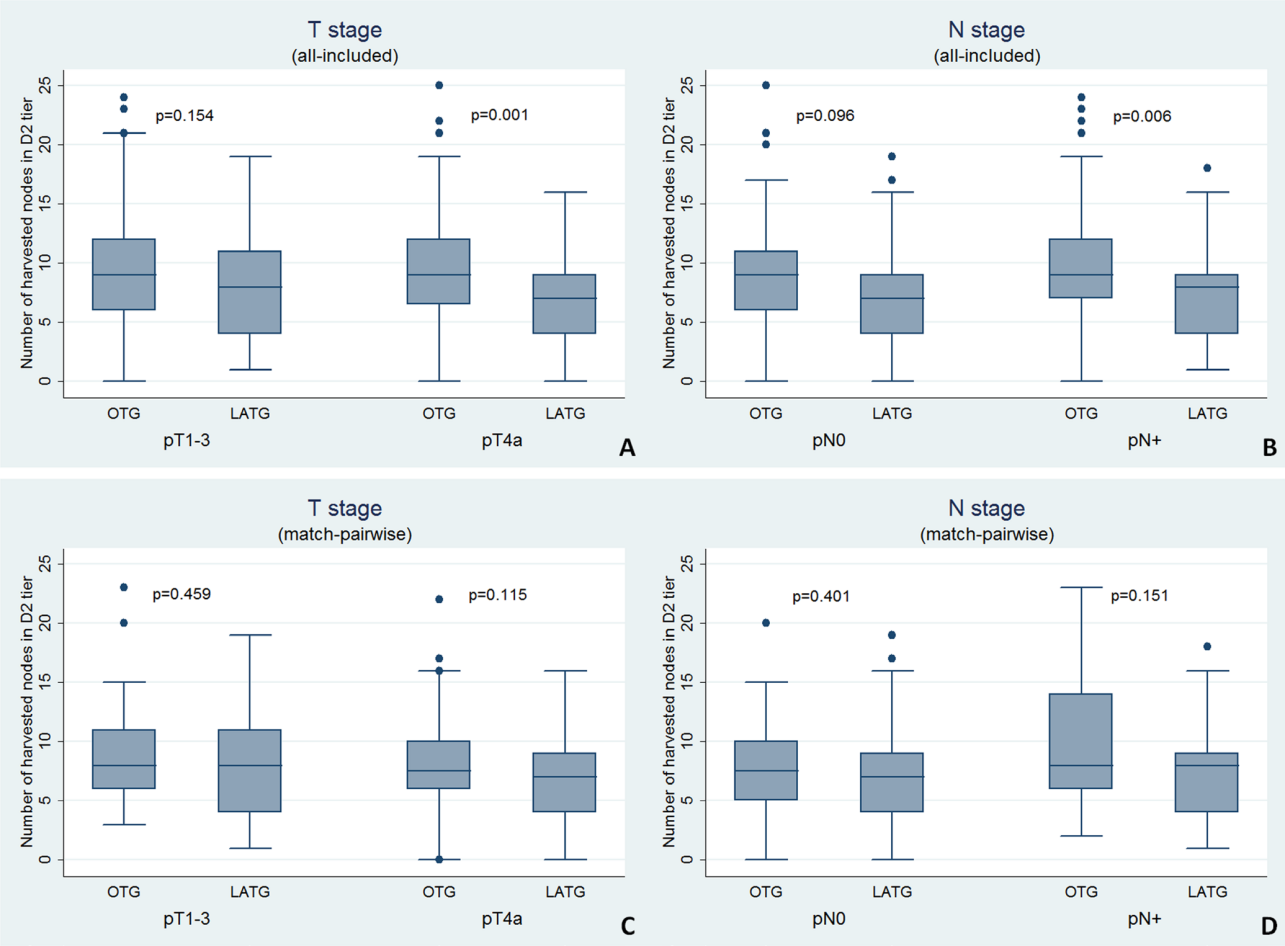
T stage and N stage specific numbers of harvested nodes in D2 tier in (**A**, **B**) all-included analysis and (**C**, **D**) match-pairwise analysis (Mann-Whitney *U* test).

The LATG group had longer operation time but harvested fewer lymph nodes for pathological examination compared with the OTG group (Table 2). However, after matching the pTNM stage, the number of harvested nodes was not significantly different between the two groups (*p* = 0.777). The proportion of D2/D2+ dissection was comparable between the two groups. The number of harvested nodes in specific stations of D2 dissection was almost similar between the two groups in the matched pairwise analysis (Table 3); statistical significance was noted for minor differences in station Nos. 9, 19 and 20. In the all-included analysis, the LATG procedure harvested fewer D2 tier nodes compared with OTG (*p* = 0.002) among pT4a or pN+ diseases (Figure 2). This difference might be introduced through the stage imbalance because it disappeared after matching. The reconstruction pattern of simple Roux-en-Y esophagojejunostomy was preferable in both groups, with the exception of 6.0% patients in the OTG group who underwent Roux-en-Y esophagojejunostomy with a pouch (Table 2).

**Table 3:** Numbers of harvested lymph nodes by specific stations of standard D2 gastrectomy

Nodal stations	All OTG Mean ± SD	Matched OTG Mean ± SD	LATG Mean ± SD	*P*_1_ value*	*P*_2_ value*
No. 1	2.13 ± 2.28	2.00 ± 2.17	1.97 ± 1.68	0.592	0.927
No. 2	2.30 ± 2.00	2.28 ± 1.94	2.02 ± 2.06	0.328	0.465
No. 3	6.44 ± 5.74	6.50 ± 4.86	6.19 ± 4.42	0.778	0.734
No. 4d	3.58 ± 3.42	3.15 ± 3.18	3.56 ± 2.41	0.973	0.401
No. 4sa	1.07 ± 1.95	0.66 ± 1.12	1.04 ± 1.45	0.896	0.120
No. 4sb	1.17 ± 1.86	0.85 ± 1.45	1.03 ± 1.50	0.593	0.492
No. 5	0.92 ± 1.24	0.81 ± 1.03	0.61 ± 0.90	0.059	0.249
No. 6	4.01 ± 3.22	4.02 ± 3.26	3.63 ± 2.63	0.386	0.471
No. 7	3.29 ± 2.52	2.97 ± 2.48	3.34 ± 2.20	0.875	0.364
No. 8a	1.79 ± 1.27	1.49 ± 1.05	1.81 ± 1.26	0.908	0.123
No. 9	2.36 ± 1.95	2.91 ± 2.29	2.15 ± 1.87	0.459	0.046
No. 10	1.12 ± 1.48	1.07 ± 1.82	0.65 ± 1.12	0.029	0.147
No. 11p	1.99 ± 1.66	2.00 ± 1.89	2.05 ± 1.91	0.812	0.883
No. 11d	1.22 ± 1.15	1.22 ± 1.26	0.92 ± 1.03	0.085	0.184
No. 12a	0.91 ± 1.12	0.70 ± 1.04	0.74 ± 1.18	0.300	0.858
No. 19	0.14 ± 0.45	0.13 ± 0.40	0.50 ± 0.91	<0.001	0.014
No. 20	0.74 ± 1.29	0.22 ± 0.51	0.51 ± 0.61	0.312	0.021
No. 110	0.55 ± 0.87	0.53 ± 0.92	0.38 ± 0.87	0.535	0.665
No. 111	0	0	0	−	−
D1 tier	20.15 ± 10.66	20.19 ± 9.74	20.25 ± 8.56	0.942	0.970
D2 tier	9.59 ± 4.54	9.00 ± 4.71	7.70 ± 4.33	0.002	0.092

Abbreviations: SD, standard deviation.

*One-way ANOVA test. *P*_1_ values for all-included comparisons; *P*_2_ values for match-pairwise comparisons.

### Survival outcomes

**Figure 3:**
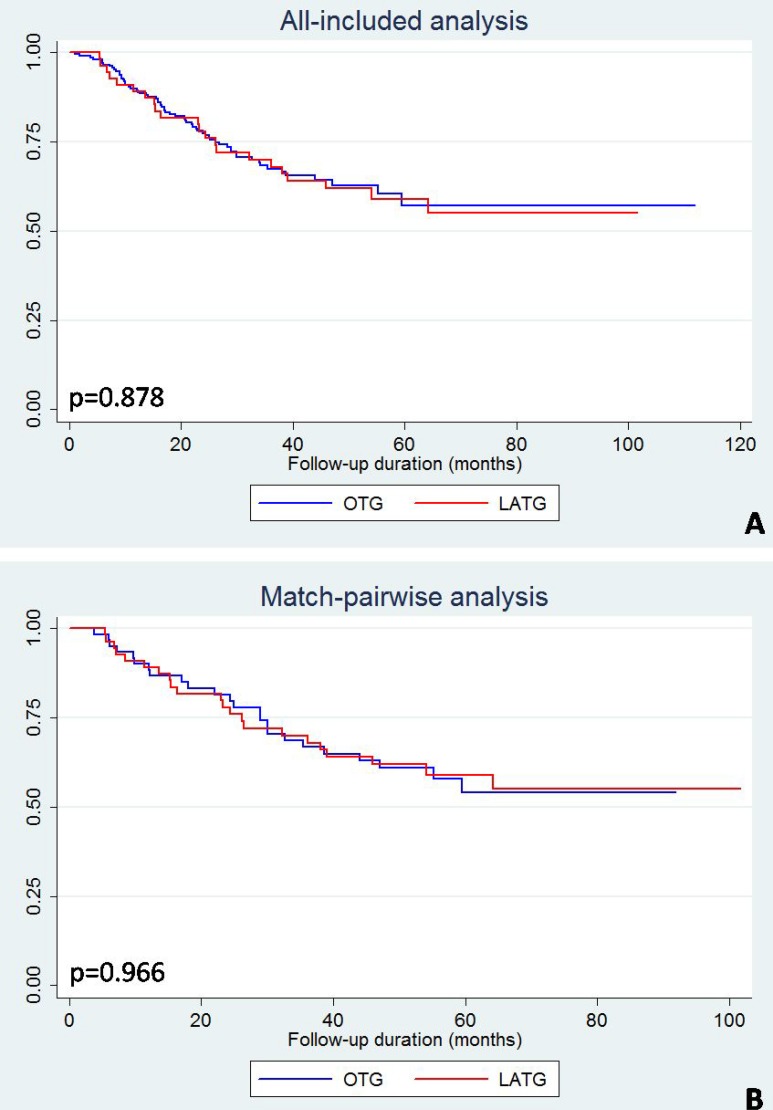
Kaplan-Meier curves of LATG and OTG in (**A**) all-included analysis and (**B**) match-pairwise analysis.

**Figure 4:**
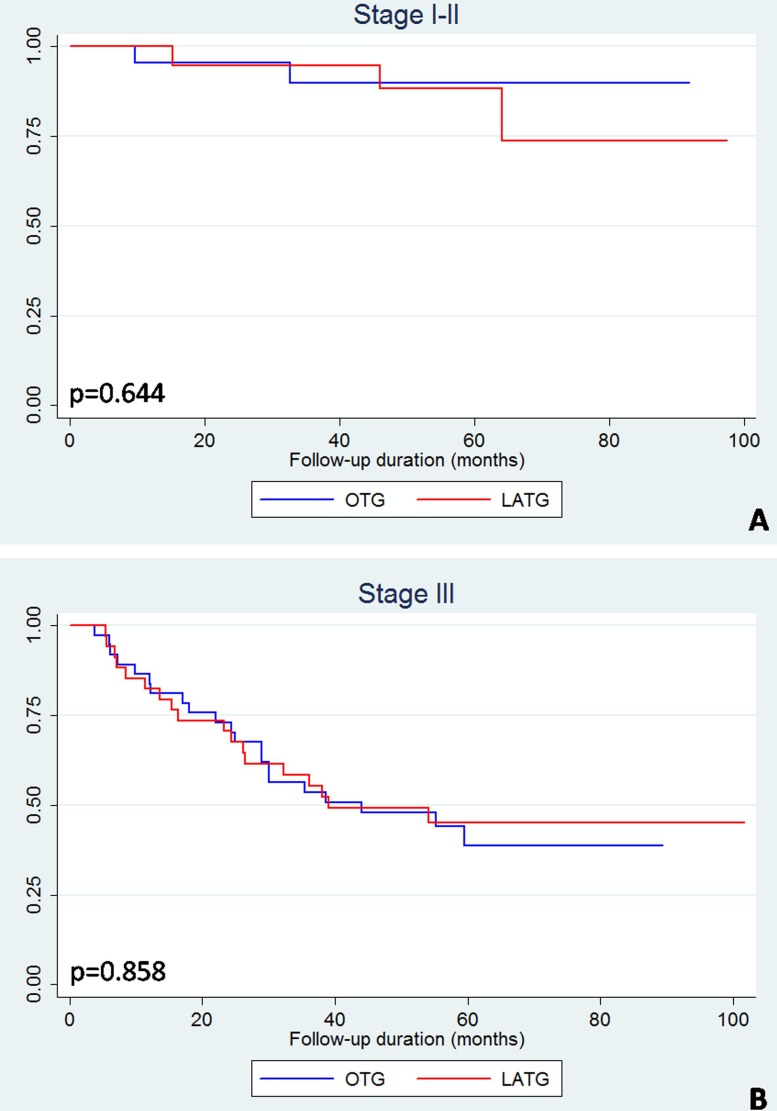
Kaplan-Meier curves of LATG and OTG among match-pairwise patients, stratified as stage I-II and stage III

**Figure 5:**
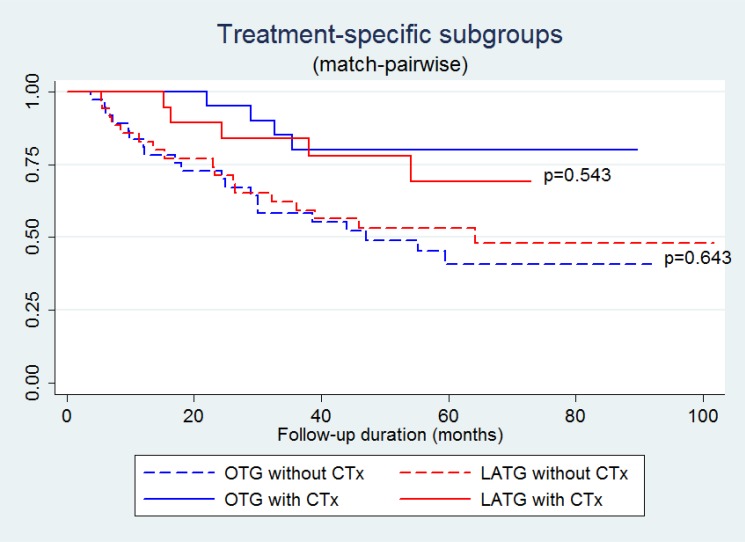
Kaplan-Meier curves of treatment-specific subgroups (surgical procedure and adjuvant chemotherapy, CTx) among match-pairwise patients

The all-included and match-pairwise comparison did not reveal differences in the Kaplan-Meier curves between the LATG and OTG groups (Figure 3). The subgroup analyses based on pairwise patients were performed by stratifying the patients into stage I–II and stage III subgroups. The stage-specific Kaplan-Meier curves of LATG and OTG were also not significantly different (Figure 4). Similarly, regardless of adjuvant chemotherapy, the Kaplan-Meier curves of LATG and OTG were comparable (Figure 5). Among all the observations, the 5-year survival rates of OTG and LATG groups were 53.5% (45.3%-61.6%) and 61.1% (48.1%-74.1%), respectively, whereas the median survival times were not achieved (Table 4). In matched analysis, stage III subgroups of the two groups achieved median survival times of 38.7 (22.0–56.6) months and 38.5 (16.4–64.4) months, respectively (Table 4). Both univariate and multivariate analyses demonstrated that LATG was not a risk factor for OS compared with OTG (Table 5). Additionally, multivariate analysis demonstrated that pT4a, pN+ and lack of adjuvant chemotherapy were independent prognostic factor for worse survival among pairwise matched patients.

**Table 4:** Survival rates and median survival times

Subsets	OTG			LATG		
	3-y SR, % (95% CI)	5-y SR, % (95% CI)	MST, months (IQR)	3-y SR, % (95% CI)	5-y SR, % (95% CI)	MST, months (IQR)
**All included observations**	70.3 (63.8–76.7)	53.5 (45.3–61.6)	NR	75.4 (64.9–85.9)	61.1 (48.1–74.1)	NR
TNM stage						
I-II	94.7 (88.9–100)	91.2 (81.6–100)	NR	96.3 (89.2–100)	90.0 (76.9–100)	NR
III	61.3 (52.7–69.9)	44.2 (34.2–54.2)	NR	62.2 (46.5–77.8)	45.5 (28.5–62.4)	38.5 (16.4–64.4)
Adjuvant chemotherapy						
Yes	77.2 (68.6–85.8)	60.0 (47.1–72.9)	NR	85.7 (70.7–100)	68.8 (46.0–91.5)	NR
No	64.1 (54.8–73.3)	51.2 (40.6–61.7)	NR	70.5 (57.0–83.9)	57.9 (42.2–73.6)	NR
**Matched observations**	70.3 (59.1–81.5)	47.8 (33.4–62.3)	NR	75.4 (64.9–85.9)	61.1 (48.1–74.1)	NR
TNM stage						
I-II	92.3 (82.1–100)	87.5 (71.3–100)	NR	96.3 (89.2–100)	90.0 (76.9–100)	NR
III	54.1 (38.0–70.1)	27.6 (11.3–43.9)	38.7 (22.0–56.6)	62.2 (46.5–77.8)	45.5 (28.5–62.4)	38.5 (16.4–64.4)
Adjuvant chemotherapy						
Yes	81.8 (65.7–97.9)	60.0 (29.6–90.4)	NR	85.7 (70.7–100)	68.8 (46.0–91.5)	NR
No	64.3 (49.8–78.8)	44.4 (28.2–60.7)	38.7 (17.0–59.4)	70.5 (57.0–83.9)	57.9 (42.2–73.6)	NR

Abbreviations: CI, confidence interval; IQR, interquartile range; MST, median survival time; NR, not reached; SR, survival rate.

**Table 5:** Univariate and multivariate analysis on prognostic factors

Variable (reference)	All patients		Match-pairwise patients	
	Univariate	Multivariate^#^	Univariate	Multivariate^#^
	HR (95% CI)	HR (95% CI)	HR (95% CI)	HR (95% CI)
Age (≥ 65y vs. < 65y)	1.33 (0.85–2.07)	1.28 (0.80–2.06)	0.95 (0.49–1.84)	1.01 (0.49–2.10)
Sex (female vs. male)	1.09 (0.65–1.81)	0.85 (0.50–1.49)	1.49 (0.74–3.00)	1.08 (0.49–2.39)
Tumor size (≥ 5cm vs. < 5cm)	**2.88 (1.62–5.11)**	**1.84 (1.01–3.35)**	**3.21 (1.50–6.88)**	1.99 (0.89–4.44)
Tumor site (M/ML vs. EGJA/U/UML)	1.03 (0.67–1.58)	1.03 (0.66–1.63)	0.83 (0.46–1.49)	0.88 (0.46–1.69)
Macroscopic type (3–4 vs. 0–2)	1.50 (0.98–2.30)	0.91 (0.58–1.43)	1.38 (0.77–2.46)	0.96 (0.51–1.81)
Differentiation (G3-4 vs. G1-2)	1.71 (0.91–3.21)	1.69 (0.87–3.29)	1.52 (0.68–3.40)	1.48 (0.60–3.67)
T stage (T4a vs. T1-3)	**3.28 (1.86–5.82)**	**2.09 (1.15–3.80)**	**5.23 (2.07–13.24)**	**3.09 (1.17–8.13)**
N stage (N+ vs. N0)	**13.78 (3.39–56.06)**	**10.10 (2.43–42.00)**	**10.20 (2.47–42.08)**	**6.13 (1.42–26.47)**
M stage (M1 vs. M0)	**2.44 (1.22–4.87)**	1.58 (0.76–3.26)	2.96 (0.71–12.27)	0.95 (0.20–4.49)
Perineural invasion (postive vs. negative)	0.68 (0.33–1.40)	0.48 (0.23–1.04)	0.75 (0.27–2.10)	0.39 (0.13–1.14)
Vessel invasion (positive vs. negative)	1.84 (1.13–3.02)	1.71 (1.01–2.89)	1.76 (0.93–3.35)	1.30 (0.65–2.61)
Surgical procedure (LATG vs. OTG)	1.04 (0.64–1.70)	1.16 (0.68–1.97)	0.99 (0.55–1.76)	1.29 (0.68–2.44)
Adjuvant chemotherapy (without vs. with)	**2.23 (1.41–3.52)**	**2.32 (1.45–3.72)**	**2.88 (1.39–5.97)**	**2.75 (1.29–5.87)**

Abbreviations: CI, confidence interval; HR, hazard ratio.

^#^ Model without selection.

## DISCUSSION

This retrospective case-control study found that LATG was as safe as OTG without increasing postoperative morbidity and mortality. The number of harvested lymph nodes in LATG was reduced comparedwith OTG possibly due to a stage imbalance between the two procedures. Approximately all patients underwent D2/D2+ dissection, and the average number of harvested nodes was greater than 30 in the LATG procedure. The overall survival outcomes were comparable between LATG and OTG procedures. The sensitivity analysis based on matched pairwise case-control comparison additionally validated these findings.

Currently, the JGCA only recommends laparoscopic distal gastrectomy as an optional treatment for cStage-I cancer by experienced and in-house certified surgeons [17], which was supported by the JCOG-0703, JCOG-0912 and KLASS-01 trials [18–20]. However, the JGCA still restricts laparoscopic gastrectomy as an investigational treatment for advanced diseases or total gastrectomy given the lack of prospective evidence [17]. The CLASS-01 trial was the first RCT under the Chinese Laparoscopic Gastrointestinal Surgery Study (CLASS) group and reported the surgical safety of laparoscopic D2 dissection among advanced distal gastric cancers [11]. In addition, long-term results of the ongoing JLSSG-0901 and KLASS-02 trials for advanced diseases are also expected [21, 22]. The CLASS-02 [23] and JCOG-1401 [24] trials were launched and aim to assess the feasibility, safety and surgical oncologic outcomes of laparoscopic total gastrectomy. Its short-term and long-term results are expected to provide better evidence on the evaluation of the laparoscopic technique in total gastrectomy. Laparoscopic total gastrectomy has received considerable attention to date but is still not recommended in general practice.

We need recognize that the technical complexity and difficulties associated with LATG are a major concern regarding the safety and surgical oncology for gastric cancer patients. LATG is technically difficult compared with OTG and therefore associated with longer operation duration and fewer harvested nodes. However, after matching the pTNM stage, the numbers of harvested nodes did not remain different between the two groups. These findings demonstrated that the fewer nodes harvested might be confounded by the imbalance of N stage, but not associated with the procedures. Nevertheless, compared with laparoscopic distal gastrectomy, lymphadenectomy at the splenic hilar, hiatus, and lower mediastinum is more difficult [25–27], requires a longer operation duration, and is associated with a risk of splenic vein or pleura parietalis injury. Esophagojejunostomy is relatively difficult when performed via mini-incision or completely through laparoscopy [28]. JGCA guidelines commented that laparoscopic total gastrectomy might be associated with an increased risk of postoperative complications during the first year of performance [17]. Song, et al. found that D2 dissection was common in LATG procedures and also safe even during a surgeon's early experience with the technique [29]. Nevertheless, we suggest that candidate surgeons for laparoscopic total gastrectomy should be experienced and skillful in laparoscopic distal gastrectomy. LATG should be restricted during the learning curve of laparoscopic distal gastrectomy. Furthermore, the learning curve of LATG also requires a high level of attention. The practice should progress from low BMI to high BMI patients and also from D1 to D2/D2+ lymphadenectomy [8]. Establishing standardized surgical procedures for LATG is helpful for those learning the technique [30].

Additionally, according to Japanese guidelines on gastric cancer treatment, No. 10 station dissection is required in a D2 total gastrectomy [17, 31]. The thoroughness of lymph node dissection should not be different between open and laparoscopic gastric cancer surgeries; however, No. 10 station dissection is complex and challenging in both procedures. To remove the lymphatic fatty tissue from spleen hilum as completely as possible, Huang CM, et al. skeletonized the spleen hilar vessels in laparoscopic total gastrectomy using a fixed maneuver [32]. The ongoing CLASS-04 Chinese study aimed to evaluate the feasible and safety of laparoscopic spleen-preserving No. 10 lymph node dissection [33]. However, another controversy for No. 10 station involves whether it is necessary to dissect the spleen in a prophylactic manner. In the Japanese JCOG-0110 trial, splenectomy did not improve survival for total gastrectomy for proximal gastric cancer not invading the greater curvature [34, 35]. Therefore, current opinions tend to modify the D2 total gastrectomy without mandatory No. 10 dissection among patients exhibiting that pattern; however, the No. 10 station is still defined as a regional node. If the greater curvature is invaded, the metastasis rate of No. 10 station was as great as 16% [36], and No. 10 station dissection is indicated.

On the other hand, given its minimally invasive nature, LATG might lead to faster postoperative recovery and reduced postoperative hospitalization. The advantages of laparoscopic distal gastrectomy for short-term outcomes has been approved by the meta-analysis based on RCTs [37]. Despite the lack of prospective trials, some retrospective evidence has supported the feasibility of laparoscopic total gastrectomy. Ramagen, et al. and Shu, et al. found that laparoscopic D2 total gastrectomy reduces the operation time, time for refeeding and hospitalization compared with open surgery [38, 39]. The present findings are generally consistent with previous retrospective studies and meta-analyses [40–43]. Adjuvant chemotherapy is accepted as an essential part of multidisciplinary treatment for gastric cancer and associated with improved prognosis [44, 45]. Given earlier initiation of adjuvant chemotherapy leading to better survival outcome, the minimally invasive nature of LATG may decrease the timing interval between operation and adjuvant chemotherapy [46]. Moreover, we should pay attention to elderly patients (≥70 years) who are more vulnerable to severe complications after laparoscopic total gastrectomy, such as anastomosis leakage [47]. Beyond the minimally invasive benefit, the age associated risks should be cautiously considered in LATG candidate selection.

Additionally, the long-term survival outcome was comparable between laparoscopic and open total gastrectomy in a meta-analysis [14, 42]. A case-matched controlled prospective analysis demonstrated similar and acceptable cumulative incidence of recurrence and disease-free or overall survival rates between laparoscopic and open total gastrectomy [39]. Given the retrospective nature of the present study, the OTG group was composed of patients with more advanced diseases compared with the LATG group, whereas the survival outcome was not significantly different. In this case, we questioned whether the results inferred better survival based on the OTG procedure. Further pairwise matched sensitivity analysis, including stage-specific and treatment-specific subgroup analyses, eliminated the doubt induced from the imbalance of baseline stages. A possible explanation was that experienced surgeons could complete LATG with both comparable proportions of D2/D2+ dissection and greater than 15 nodes harvested compared with the OTG procedures. Therefore, the current evidence supported the advantage of the minimally invasive nature and equivalent surgical outcome and survival; however, the robustness was limited to some extent.

There were several limitations of the present study. First, the retrospective design might introduce some selection bias and performance bias. To limit type II error, we performed sensitivity analyses in a pairwise manner to overcome the imbalance at the baseline. However, the propensity score matching method is a suitable method to fully consider confounders; however, only the simple matching method was applied in the present study. In addition to the major prognostic factor TNM stage, a minor risk of residual selection bias, such as BMI and tumor size, was noted (Table 1). Second, the single center dataset with the limited sample size might impair the test power. The riskof false negative results among OS comparisons were not omitted. Before a definitive conclusion is reached, high-quality randomized controlled trials with sample size calculations are required to judge the long-term survival of the LATG procedure. Third, the median follow-up duration was less than five years. The differences in survival outcomes between two procedures might be underestimated. A longer observation and further repetitive analyses are required. Finally, the disease-free survival and recurrence patterns not specified due to incomplete data. Although the OS outcome was not different between two procedures, the risk of recurrence or metastasis from LATG was unclear.

In conclusion, LATG by experienced surgeons has comparable short-term surgical outcomes and long-term survival outcomes compared with OTG for gastric cancer patients. However, high-quality large-scaled RCTs are necessary before confirmative judgment and recommendation as an optional treatment in general practice.

## MATERIALS AND METHODS

### Study design

A retrospective case-control study was conducted with additional matching case-control analyses. This study aimed to compare the short-term and long-term outcomes between LATG and OTG groups. The hypothesis was H_0_ = the risks of postoperative complication, mortality, and survival outcomes of LATG are comparable to OTG.

### Ethics

The laparoscopic gastric cancer surgery technique and the collection of medical information from the Surgical Gastric Cancer Patient Registry in West China Hospital were approved by the Biomedical Ethical Committee of West China Hospital, Sichuan University [48]. The participants did not provide written informed consent due to the nature of the retrospective study; however, the patients’ records were anonymized and de-identified prior to analyses by researchers. Other researchers in this study did not have access to the patients’ identifying information or records prior to anonymization. The study complied with the World Medical Association Declaration of Helsinki regarding the ethical conduct of research involving human subjects.

### Patient information

Data retrieval was based on the Surgical Gastric Cancer Patient Registry in West China Hospital [15, 49, 50]. Eligible patients with complete medical records were identified between June 1, 2006 and December 31, 2015 for the analyses on morbidity and mortality. The survival analysis was performed within the period from June 1, 2006 to December 31, 2014 with at least one-year follow-up. The inclusion criteria of LATG candidates were as follows: 1) patients with histologically proven gastric adenocarcinomas; 2) patients underwent curative total gastrectomy through either laparoscopy-assisted or conventional open approach; 3) aged 18 to 80 years. The exclusion criteria of LATG were as follows: 1) combined resection of spleen, pancreatic tail, or transverse colon; 2) neoadjuvant chemotherapy or radiotherapy; 3) T4b disease; 4) malignancy history; 5) pregnancy. The candidates eligible for LATG were informed to select open or laparoscopic procedures preoperatively. Procedure selection was depended on the personal willingness of patients. In the present study, those patients who underwent OTG were identified in the same period and with the same eligibility criteria.

### Surgery procedures

**Figure 6:**
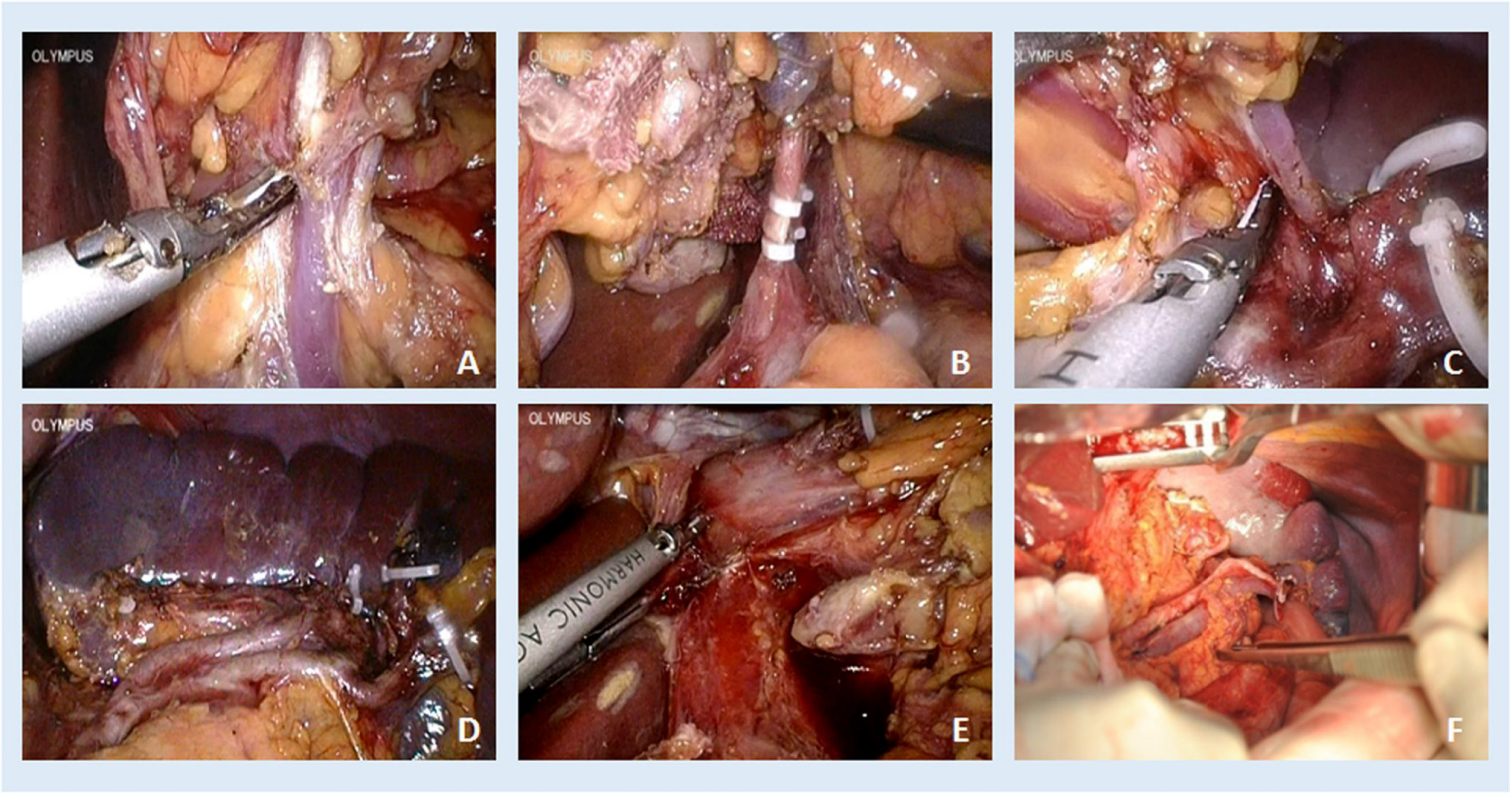
Illustration of LATG-D2 (**A**) No. 6 station, (**B**) No. 7, 8a, 9, 11p, (**C**) No. 4sb, 11d, (**D**) No. 10, (**E**) No. 1 and hiatus, and OTG-D2 (**F**) No. 11d, 10.

All operations of analyzed patients were performed by an experienced surgeon (J. K. Hu) (Figure 6). Lymphadenectomy performed was according to the treatment guidelines of Japanese Gastric Cancer Association (JGCA) with a modification [51]. For tumors located at ML/M sites, nodes from No. 1, 2, 3a, 3b, 4, 5, 6, 7, 8a, 9, 10, 11p, 11d, 12a, 19, and 20 groups were dissected. Additionally, for tumors located at U/EGJ sites, the above nodes plus No. 110 and 111 groups were dissected using the transhiatal approach. The definitions of lymphadenectomy were classified as D1/D1+ and D2/D2+ [17]. The D1 tier nodes included No. 1, 2, 3, 4d, 4sa, 4sb, 5, 6 and 7 stations, whereas the D2 tier nodes included No. 8a, 9, 10, 11p, 11d, 12a, 19, 20, 110 and 111 stations. Reconstruction patterns included simple Roux-en-Y esophagojejunostomy or Roux-en-Y esophagojejunostomy with jejunal pouch. In the LATG procedure, mobilization and lymphadenectomy were completed by laparoscopy, and gastrectomy and reconstruction were completed by laparotomy through a middle-line mini-incision (approximately 10 cm).

### Pathology

The postoperative pathological assessment was performed in a peer review manner by two independent pathologists in the Department of Pathology, West China Hospital [50, 52]. The surgical samples were 10% neutral formalin-fixed for 8 hours and then dehydrated. The paraffin-embedded blocks were prepared in sections. Hematoxylin and eosin staining was used to evaluate tumor differentiation, infiltration depth, and lymph node metastasis. The pathological classification and staging were performed according to the JGCA classification and the AJCC 7th TNM system [31, 53].

### Follow-up

Regular follow-up (Supplementary Table 1) was suggested and performed every three months in the postoperative 1st year and every six months in the postoperative 2nd–5th years. Incident outpatient visits were also recorded. The follow-up information was updated in the registry database every half a year. Overall survival (OS) was estimated at each follow-up. The last update of follow-up information was Jan 1, 2016.

### Outcome measurement

The primary outcome was OS. The secondary outcomes were postoperative complication incidence and severity (by the Clavien-Dindo classification) and surgical parameters, including operation duration, blood loss, number of harvested lymph nodes, and postoperative hospital stay.

### Statistics

To analyze baseline characteristics and short-term results, the ranked variables were compared using the Mann-Whitney *U* test, and continuous variables were compared using the Mann-Whitney *U* test or one-way ANOVA, where applicable. Categorical variables were compared with Pearson's Chi-square test or Fisher's exact test. Kaplan-Meier curves and log-rank test were used to compare the OS between LATG and OTG groups. Univariate and multivariate analyses were performed by Cox proportional hazards model with Breslow method for ties. Hazard ratios (HRs) with 95% confidence intervals (CIs) were estimated. Cox models in multivariate analyses were adjusted for those potential confounders (clinicopathologic features, surgical and adjuvant treatment) without selection. Sensitivity analyses were performed in a manner of additional 1:1 matching case-control study. Matching factors included age (difference ≤ 5 years), sex, operation year, and pathological TNM stage. The same analyses were performed again among the matched pairwise patients and for all-included analyses. A *p* value less than 0.05 was considered statistically significant. STATA/SE 12.0 software was used for statistical analysis [54].

## SUPPLEMENTARY TABLE


