# Supplementary material for: Comparisons of short-term and survival outcomes of laparoscopy-assisted versus open total gastrectomy for gastric cancer patients
Source: Oncotarget. 2017 Apr 10;8(32):52366–80. doi: 10.18632/oncotarget.17019 (PMC5581035; doi:10.18632/oncotarget.17019)
Supplement: Supplementary file 1 [file oncotarget-08-52366-s001.pdf]

Comparisons of short-term and survival outcomes of laparoscopy-assisted versus open total gastrectomy for gastric cancer patients

Supplementary Materials

Supplementary Table 1: Recommended postgastrectomy follow-up program

Postoperative month	03	06	09	12	18	24	30	36	42	48	54	60
OS status	×	×	×	×	×	×	×	×	×	×	×	×
CT[a]		×		×	×	×		×		×		×
Ultrasound	×	×		×		×		×		×		×
Endoscopy				×		×		×		×		×
Serology[b]	×	×		×		×		×		×		×
Others[c], and the above	Relevant symptom or PE sign driving at any time											

[a] Enhanced MDCT for abdomen, non-enhanced CT for thorax.
[b] BRT and SMA, mandatroy; CA724, CEA, and CA199, selected.
[c] PET/CT, bone scan, brain MRI.
